# Rapid Progression of a Cavernous Sinus Dural Arteriovenous Fistula From Borden Type I to Type II: A Case Report

**DOI:** 10.7759/cureus.90050

**Published:** 2025-08-14

**Authors:** Satoshi Horiguchi, Yoshinori Maki, Yuto Mitsuno, Kota Nakajima, Ryosuke Nishi

**Affiliations:** 1 Department of Neurosurgery, Nagahama City Hospital, Nagahama, JPN; 2 Department of Neurosurgery, Hikone Chuo Hospital, Hikone, JPN; 3 Department of Neurosurgery, Kyoto University Graduate School of Medicine, Kyoto, JPN

**Keywords:** borden classification, cavernous sinus dural arteriovenous fistula, cortical venous reflux, neuroimaging and neurointervention, rapid progression, transvenous embolization

## Abstract

Cavernous sinus dural arteriovenous fistulas (CSdAVFs) are typically considered benign when classified as Borden type I, which is characterized by the absence of cortical venous reflux (CVR). These lesions are often managed conservatively with long-term follow-up imaging. Although progression to higher-risk types involving CVR can occur, it is generally gradual.

A 74-year-old woman presented with recurrent diplopia due to a right abducens nerve palsy. Digital subtraction angiography revealed Borden type I CSdAVFs with venous drainage exclusively via the bilateral inferior petrosal sinuses, without evidence of CVR. Conservative management with follow-up MRI was initially planned. However, three months later, the patient developed new-onset pulsatile tinnitus and conjunctival injection. Follow-up imaging demonstrated newly developed CVR and altered venous drainage patterns, consistent with progression to Borden type II. Transvenous embolization resulted in the complete resolution of symptoms and a favorable clinical outcome (modified Rankin Scale score of 0).

This case highlights the potential for rapid progression of CSdAVFs, even in lesions initially classified as Borden type I. Vigilant clinical monitoring and prompt re-imaging are essential when new symptoms emerge, even within a short interval following diagnosis.

## Introduction

Dural arteriovenous fistulas (dAVFs) involving the cavernous sinus represent a distinct subset of intracranial vascular malformations, characterized by abnormal arteriovenous shunting within the dural leaflets of the cavernous sinus. These lesions manifest with a range of ophthalmologic and neurologic symptoms due to cranial nerve involvement and altered venous drainage patterns [1]. The Borden classification system is widely used to stratify dAVFs based on their venous drainage patterns and associated risk of hemorrhage [2]. Borden type I lesions, which drain exclusively into the dural venous sinuses without cortical venous reflux (CVR), are generally considered benign and are often managed conservatively [3,4]. However, progression to higher-risk types with the development of CVR has been documented, typically unfolding over a prolonged clinical course [3,5,6]. Rapid progression from Borden type I to type II or III within a short interval is rare and carries significant implications for clinical monitoring and management [1,3,6]. We report a rare case of an angiographically confirmed Borden type I cavernous sinus dural arteriovenous fistula (CSdAVF) that rapidly progressed to Borden type II within three months.

## Case presentation

**Figure 1:**
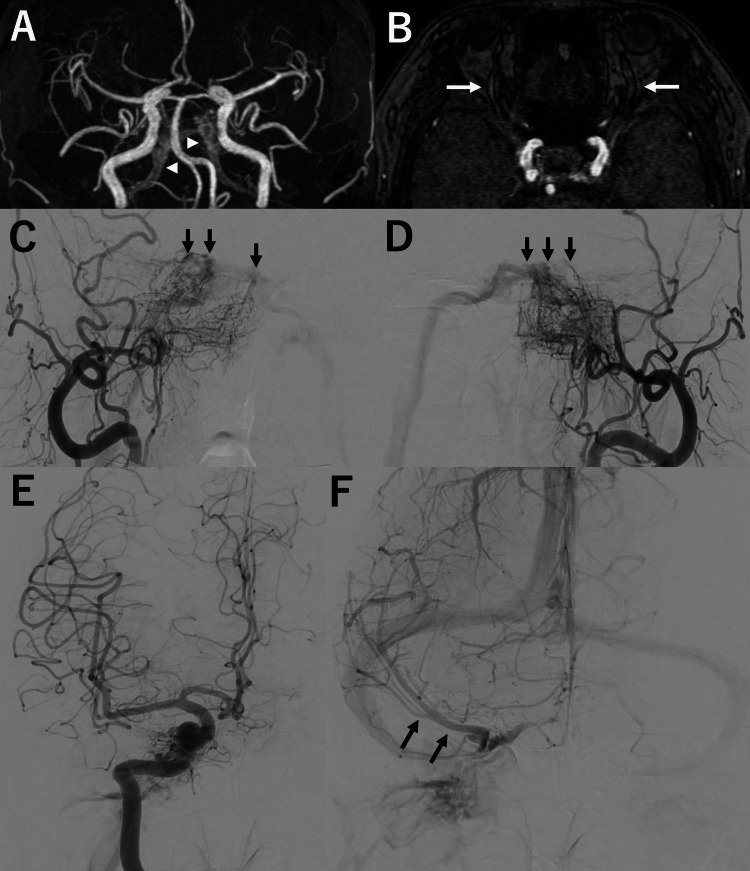
Initial radiological findings MRA: magnetic resonance angiography; CS: cavernous sinus; IPS: inferior petrosal sinus; dAVF: dural arteriovenous fistula; DSA: digital subtraction angiography; SMCV: superficial middle cerebral vein (A) MRA shows abnormal flow signals in the CS region extending into the bilateral IPSs (arrowheads), suggesting a dAVF. (B) A time-of-flight axial image demonstrating normal caliber of the bilateral superior ophthalmic veins (arrows). (C, D) DSA of the right (C) and left (D) external carotid arteries confirms the presence of a CSdAVF, with the shunt point indicated by arrows and primary venous drainage through the bilateral IPSs. (E, F) Right internal carotid artery angiography in the arterial phase reveals venous drainage into the right IPS, while the venous phase shows normal flow into the right SMCV (F, arrows)

A 74-year-old woman with a medical history of hypertension and dyslipidemia but no other significant comorbidities experienced transient diplopia three months prior to presentation, which resolved spontaneously. However, persistent diplopia recurred two weeks before evaluation, prompting further investigation. Magnetic resonance angiography (MRA) revealed abnormal flow signals in the cavernous sinus region (Figures 1A, 1B), raising suspicion for a dAVF.

Neurological examination showed diplopia that worsened during rightward gaze, consistent with a right abducens nerve palsy. Digital subtraction angiography (DSA) confirmed a dAVF involving the cavernous sinus (Figures 1C, 1D). Arterial feeders included the bilateral middle meningeal and ascending pharyngeal arteries. Venous drainage occurred exclusively via the bilateral inferior petrosal sinuses (IPSs), with no evidence of CVR or retrograde drainage into the superior ophthalmic veins (SOVs) (Figures 1E, 1F). The lesion was classified as Borden type I. Given its benign appearance, conservative management with a follow-up MRI was initially selected.

**Figure 2:**
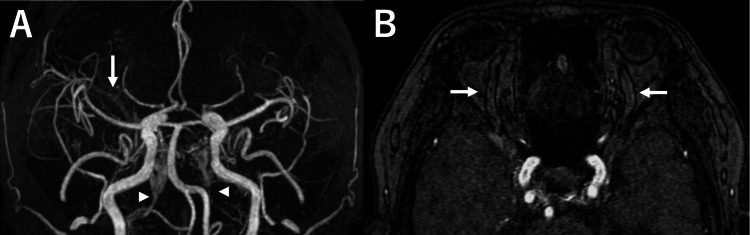
Follow-up images three months after the initial examinations MRA: magnetic resonance angiography; CVR: cortical venous reflux; IPS: inferior petrosal sinus; SOV: superior ophthalmic vein (A) MRA obtained three months after the diagnosis demonstrates an increased flow-related signal in the right Sylvian fissure (arrow), suggestive of newly developed CVR. Additionally, narrowing of the right IPS and nonvisualization of the left IPS are observed (arrowheads). (B) A time-of-flight axial image shows bilateral enlargement of the SOVs (arrows)

At a follow-up visit approximately three months after the initial diagnosis, the patient reported new-onset pulsatile tinnitus that had begun two weeks earlier, and her neurological status had remained stable until that time. Bilateral conjunctival injection was also noted during the physical examination. Follow-up MRA demonstrated enlargement of the bilateral SOVs and an increased flow-related signal in the right Sylvian fissure, suggestive of newly developed CVR (Figures 2A, 2B). In addition, narrowing of the right IPS and nonvisualization of the left IPS were observed, indicating a significant alteration in venous drainage. These findings prompted reconsideration of the initial conservative approach, and endovascular treatment was recommended.

**Figure 3:**
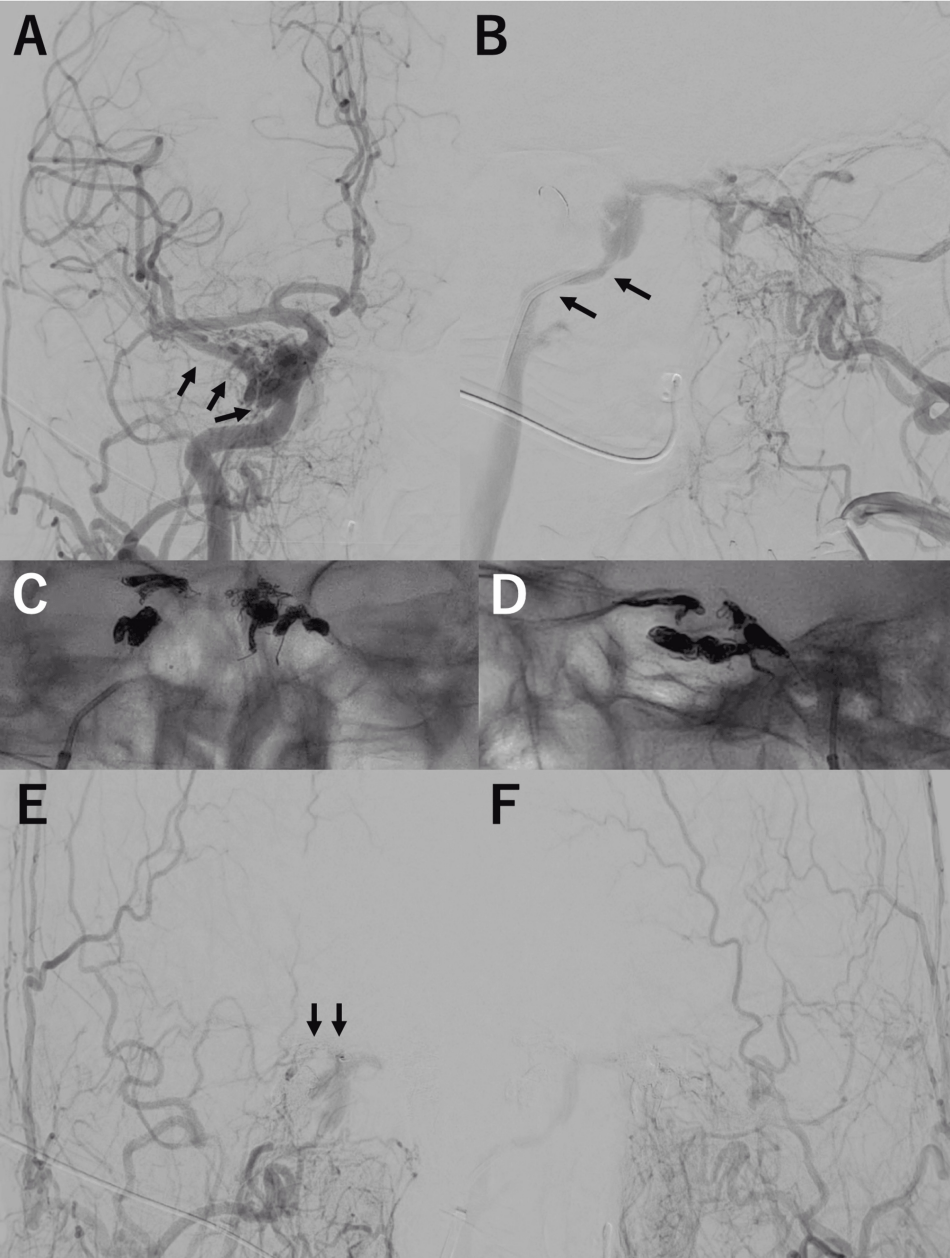
Endovascular procedure DSA: digital subtraction angiography; TVE: transvenous embolization; SOV: superior ophthalmic vein; SMCV: superficial middle cerebral vein; IPS: inferior petrosal sinus (A, B) DSA performed at the time of TVE confirmed retrograde venous drainage into the bilateral SOVs and the right SMCV (A, arrows), with persistent drainage into the right IPS (B, arrows) and occlusion of the left IPS. (C, D) Anteroposterior (C) and lateral (D) fluoroscopic views at the end of TVE demonstrate coil placement at the shunt points and at the sites of retrograde drainage into the right SMCV and the bilateral SOVs. (E, F) Post-embolization right (E) and left (F) external carotid artery angiograms demonstrate a marked reduction in shunt flow, with resolution of both cortical and ophthalmic venous reflux, although a small residual shunt remains (E, arrows)

Four months after the initial DSA, transvenous embolization (TVE) was performed via the bilateral IPSs. Intraprocedural DSA confirmed retrograde venous drainage into the bilateral SOVs and the right superficial middle cerebral vein (SMCV), along with persistent drainage into the right IPS (Figures 3A, 3B). The right IPS was stenotic, and the left IPS was obliterated. The lesion was reclassified as Borden type II. Embolization was performed in two steps: first, coil occlusion at the points of retrograde drainage into the right SMCV and bilateral SOVs, followed by target embolization of the shunt points within the cavernous sinus (Figures 3C, 3D). Post-embolization angiography demonstrated a marked reduction in shunt flow, with resolution of both cortical and ophthalmic venous reflux, although a small residual shunt persisted (Figures 3E, 3F).

**Figure 4:**
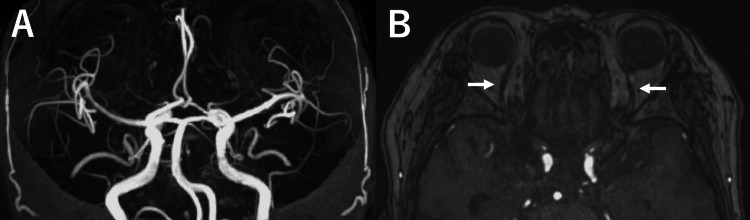
Postoperative follow-up images MRA: magnetic resonance angiography; TVE: transvenous embolization; IPS: inferior petrosal sinus; SOV: superior ophthalmic vein (A) MRA obtained three months after TVE shows disappearance of the abnormal flow signal in the cavernous sinus and bilateral IPSs. (B) A time-of-flight axial image reveals normalization of the bilateral SOVs, with a resolution of their previous enlargement (arrows)

The patient’s symptoms, including diplopia and tinnitus, resolved completely following the procedure. MRI performed the day after embolization showed residual venous flow signals in the posterosuperior portion of the right cavernous sinus. However, no flow signal was detected in the right SMCV or IPS, and the diameter of the bilateral SOVs was reduced. Four days after embolization, the patient developed right-sided ptosis and pupillary dilation due to compression of the oculomotor nerve by the embolization coils. Treatment with betamethasone and mecobalamin was initiated. One month later, the oculomotor symptoms resolved completely. Follow-up MRA demonstrated resolution of the flow signal in the cavernous sinus and bilateral IPSs (Figures 4A, 4B). Her final modified Rankin Scale score was 0.

## Discussion

This case illustrates a rare instance of rapid progression of CSdAVF from Borden type I to type II within three months. At initial diagnosis, the absence of CVR and exclusive drainage into the inferior petrosal sinuses supported a conservative management approach [2-4]. However, the onset of new symptoms, including pulsatile tinnitus and conjunctival injection, prompted clinical reassessment. Follow-up imaging revealed newly developed CVR and altered venous outflow patterns, leading to reclassification of the lesion as Borden type II [2].

Progression of dAVFs from Borden type I to more aggressive types is generally considered uncommon. Borden type I dAVFs, which lack cortical venous drainage, are typically associated with a benign clinical course. The annual conversion rate from Borden type I to higher-grade fistulas with cortical venous drainage has been reported at approximately 1.0% [7]. In one study with a mean follow-up of 5.6 years, only two patients developed symptomatic conversion to a higher-grade fistula [7]. Another study reported progression to Borden type II or III lesions in 2.2%-2.4% of patients during follow-up [8]. Furthermore, Borden type I dAVFs rarely result in intracranial hemorrhage or nonhemorrhagic neurological deficits during observation [7,9]. Spontaneous regression or symptomatic improvement has also been documented in a subset of patients, which further supports the typical benign natural history of these lesions [9].

Previous studies suggest that dAVFs may evolve due to changes in venous outflow resistance, thrombosis of venous drainage pathways, or progressive recruitment of arterial feeders [5,10]. Several factors have been associated with poor prognosis, including advanced age, presence of nonhemorrhagic neurological deficits, severe symptoms related to venous hypertension at presentation, and infratentorial lesion location [8]. Although venous ectasia and hemorrhagic presentation are known risk factors for intracranial hemorrhage in dAVFs with cortical venous drainage, these features are typically absent in Borden type I lesions [9]. Nevertheless, closer follow-up may be warranted in patients with Borden type I dAVFs who exhibit stenosis or occlusion of the affected venous sinus at initial diagnosis [9]. In this case, narrowing of the right inferior petrosal sinus and nonvisualization of the left inferior petrosal sinus likely contributed to a hemodynamic shift, resulting in the development of CVR.

Although Borden type I lesions are often monitored with annual imaging [9,11], this case underscores the importance of short-term follow-up when patients present with recurrent or new clinical symptoms. To the best of our knowledge, few reports have documented the conversion from type I to type II within such a short timeframe [1,3,6]. Notably, progression of Borden type I CSdAVF is particularly rare. Kim et al. reported four patients (4.0%; 4/99 cases) who experienced conversion from a benign to an aggressive dAVF. In one case involving a cavernous sinus lesion, loss of bruit and worsening diplopia developed over 23 months and were associated with occlusion of the left IPS and left SOV [6]. Satomi et al. described two conservatively managed cases that showed angiographic progression to lesions with cortical venous drainage during follow-up. One of these involved a cavernous sinus lesion with symptomatic worsening during a median follow-up period of 27.9 months (range: one month-17.5 years), suggesting a 2% (1/50 cases) risk of conversion to an aggressive angiographic profile for benign CSdAVFs [3].

Although the exact mechanism of IPS narrowing and occlusion in this case remains unclear, several possible contributors have been proposed in the literature. Thrombosis of venous drainage pathways may be triggered by venous stagnation, inflammatory processes, or mechanical compression. In CSdAVFs, progressive thrombosis of the IPS during conservative follow-up has been reported and is considered a key factor in the evolution toward more aggressive venous drainage patterns [3,6,10]. In our patient, the initial narrowing of the right IPS and nonvisualization of the left IPS may reflect pre-existing partial thrombosis. This process likely progressed due to sustained hemodynamic stress from the fistulous flow, ultimately resulting in altered venous drainage and the development of CVR.

In this case, the decision to perform follow-up MRA at three months was guided by the need to monitor the right abducens nerve palsy noted at presentation. While routine short-term imaging (e.g., every one to three months) cannot be recommended for all patients with Borden type I CSdAVFs, prompt re-evaluation is warranted when new or recurrent symptoms emerge. The appearance of tinnitus in this patient shortly before the scheduled follow-up underscores the value of symptom-driven imaging to detect early hemodynamic changes. This case highlights the dynamic nature of dAVFs and suggests that earlier re-imaging may be warranted, even in patients with initially benign cavernous sinus lesions, particularly when new or evolving clinical symptoms arise.

## Conclusions

CSdAVFs classified as Borden type I may undergo rapid progression to higher-risk subtypes, particularly when evolving clinical symptoms are present. This case underscores the importance of vigilant follow-up and reconsideration of imaging intervals in the management of seemingly benign dAVFs. Early detection of disease progression enables timely intervention and may help prevent complications associated with CVR.
